# Auditory attention measured by EEG in neurological populations: systematic review of literature and meta-analysis

**DOI:** 10.1038/s41598-023-47597-5

**Published:** 2023-11-29

**Authors:** Nele Vanbilsen, Sonja A. Kotz, Mattia Rosso, Marc Leman, Lisa Tedesco Triccas, Peter Feys, Lousin Moumdjian

**Affiliations:** 1 Universitair Multiple Sclerosis Centrum (UMSC), Hasselt-Pelt, Hasselt, Belgium; 2 Faculty of Rehabilitation Sciences, REVAL Rehabilitation Research Center, University of Hasselt, Agoralaan Gebouw A, 3590 Diepenbeek, Belgium; 3 Department of Neuropsychology and Psychopharmacology, Faculty of Psychology and Neuroscience, Maastricht University, Maastricht, The Netherlands; 4 Faculty of Arts and Philosophy, IPEM Institute of Psychoacoustics and Electronic Music, University of Ghent, Miriam Makebaplein 1, 9000 Gent, Belgium; 5 Université de Lille, ULR 4072 – PSITEC – Psychologie: Interactions, Temps, Emotions, Cognition, Lille, France; 6 Department of Movement and Clinical Neurosciences, Institute of Neurology, University College London, 33 Queen Square, London, UK

**Keywords:** Auditory system, Neurophysiology, Neurological disorders

## Abstract

Sensorimotor synchronization strategies have been frequently used for gait rehabilitation in different neurological populations. Despite these positive effects on gait, attentional processes required to dynamically attend to the auditory stimuli needs elaboration. Here, we investigate auditory attention in neurological populations compared to healthy controls quantified by EEG recordings. Literature was systematically searched in databases PubMed and Web of Science. Inclusion criteria were investigation of auditory attention quantified by EEG recordings in neurological populations in cross-sectional studies. In total, 35 studies were included, including participants with Parkinson’s disease (PD), stroke, Traumatic Brain Injury (TBI), Multiple Sclerosis (MS), Amyotrophic Lateral Sclerosis (ALS). A meta-analysis was performed on P3 amplitude and latency separately to look at the differences between neurological populations and healthy controls in terms of P3 amplitude and latency. Overall, neurological populations showed impairments in auditory processing in terms of magnitude and delay compared to healthy controls. Consideration of individual auditory processes and thereafter selecting and/or designing the auditory structure during sensorimotor synchronization paradigms in neurological physical rehabilitation is recommended.

## Introduction

Strategies capitalizing on sensorimotor synchronization are being applied in physical rehabilitation of walking within different neurological populations. Sensorimotor synchronization is a process where sensory and motor systems align to synchronize in time or in phase with one another^1^. These strategies are based on the coupling of bodily rhythms, such as walking with auditory rhythms such as beats found in music or metronomes. From a physical rehabilitation perspective, evidence for its use, leading to positive effects on gait has been well established in different neurological populations such as persons with Parkinson’s disease (PD)^2^, persons with multiple sclerosis (MS)^3,4^, persons with traumatic Brain Injury (TBI)^5^ and persons with stroke^6^.

Albeit these positive effects on gait, an aspect that requires elaboration during sensorimotor synchronization in neurological populations are the attentional processes that are required in order to dynamically attend the perceived temporal structure of the auditory stimuli^7,8^. To elaborate with an example of walking to auditory rhythms, in order to synchronize steps to the beats, one requires to first perceive and direct attention to the temporal information in the auditory structure in order to extract the necessary timing information. Thereafter, an attempt to lock the step in time to the beat can follow to establish sensorimotor synchronization^9^.

Given the above, it is imperative to investigate auditory attentional resources in different neurological populations as impairments in cognition, and more specifically impairments in attention are prevalent^10–13^. This work is thus situated at a meta-level of understanding selective auditory attention in different neurological populations. The rationale put forward, is that the understanding of auditory attentional processes in neurological populations as compared to healthy controls would provide guidance in titrating ingredients for the design of auditory structures suiting the attentional resources of the user. That is, with the ambition of personalized gait rehabilitation in the neurological populations.

In neuroscience, auditory oddball paradigms have been frequently used to investigate deviance processing in auditory rhythmic sequences. In these paradigms, electro-encephalography (EEG) recordings are frequently used to investigate the modulation of event-related potentials (ERPs) as brain responses to deviance^14–16^. Within these paradigms a deviant sound (also known as the target) differentiating from the standard sound is presented and participants are instructed to mentally count the number of the deviant occurrences or to react to them in terms of a button-press. As a result of this deviant sound a positive deflection around 300ms after stimulus presentation, can be detected in healthy populations, termed the P3^17^. We can measure the P3 in terms of its latency and amplitude, seen as a proxy to for attentional resources, as it reflects one’s discrimination abilities between the deviant and standard events in the auditory stimuli^18,19^.

Therefore, this systematic review was conducted to review existing literature and investigate auditory deviance processing with EEG in neurological populations and healthy participants, to better understand how possible processing delays might impact auditory stimulation in rehabilitation settings. The relevance of understanding these processes could guide to personalize the temporal structure of the auditory stimuli when applying sensorimotor synchronization strategies during neurological gait rehabilitation.

## Methodology

### Registration and search strategy

This review was registered in PROSPERO (registration number: CRD42022312932).

The search strategy was carried out in the following three databases PUBMED, Web Of Science and SCOPUS using the following terms: (oddball OR perturbations OR deviations OR novelty oddball) AND (auditory OR rhythm OR beat) AND (event-related-potentials[MeSH Terms] OR Mismatch negativity OR frequency tagging OR time-series OR electroencephalography[MeSH Terms] OR P3a OR P3b) AND (Parkinson’s disease OR multiple sclerosis OR amyotrophic lateral sclerosis OR cerebellar disorders OR Spinal cord injury OR Traumatic Brain Injury OR Stroke) NOT (Pediatrics OR Children OR Adolescents) NOT (Psychiatric disorders OR Psychological disorders) NOT (Coma).

### Selection criteria

**Figure 1:**
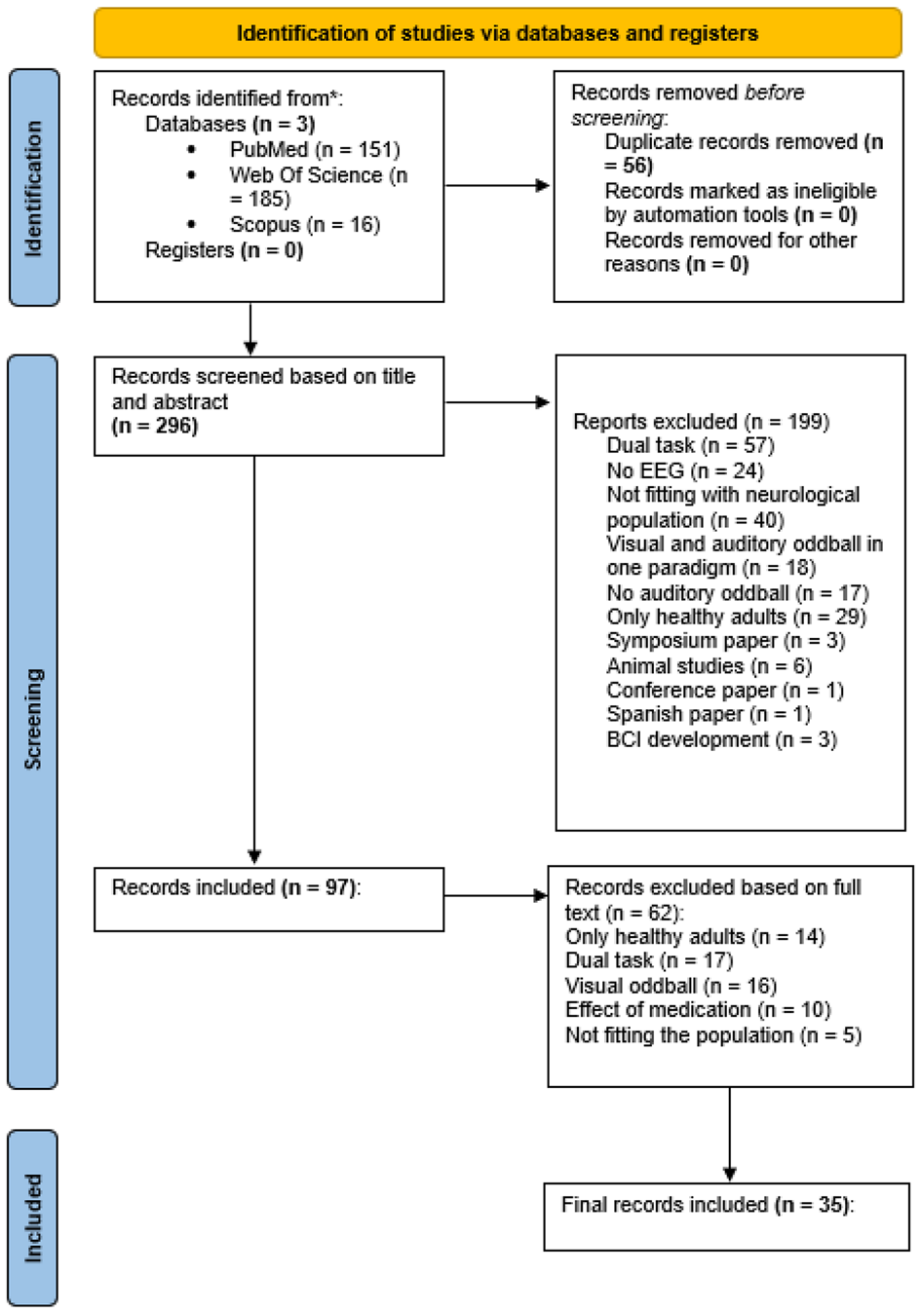
Flowchart over the search strategy and article selection process (according to the PRISMA guidelines).

Articles were selected following the PRISMA guidelines. Identification of relevant articles was performed by three independent reviewers. In case of conflict, a fourth independent reviewer was asked for screening. In total 35 articles were included. An overview of the search strategy following the PRISMA guidelines can be found in Fig. 1.

### Inclusion criteria

Cross-sectional studies (e.g. controlled trials) investigating perceptual processing of auditory rhythmic stimuli and quantified by EEG in neurological populations were included.

The neurological population included were persons with Parkinson’s disease, multiple sclerosis, amyotrophic lateral sclerosis, cerebellar disorders, spinal cord injury, stroke and traumatic brain injury, given the presence of motor or cognitive impairments in these populations. Exclusion criteria were pediatric populations, psychological or psychiatric disorders, animal studies, paradigms not using auditory stimuli, dual task paradigms where a person had to perform a motor task during the oddball paradigm such as walking, non-English papers, conference/symposium papers and paradigms using external and internal brain stimulation.

### Quality assessment

The quality assessment of the included articles was based on the STROBE checklist^20^.

### Data extraction

The following data were extracted from the selected articles: participant population (healthy or neurological disease), descriptive characteristics of the participants (age, disease information), neuropsychological information about the participants (neuropsychological test results), descriptive characteristics of the EEG paradigm used (frequency of stimuli, inter-stimulus-interval, decibels (dB) of stimuli, task instructions, stimulus length and probability of the deviant sound), electrophysiological measures (P3 ERP results (amplitude and latency) measured at Pz location).

### Data analysis

A meta-analysis comparing healthy controls and neurological populations was performed on P3 amplitude and latency data using Review Manager version 5.4.1 for a meta-analysis using random effects and 95% confidence intervals (CIs). Subgroup analyses were performed stratifying the data into neurological populations. Studies were included in the meta-analysis when P3 amplitude and latency values were provided.

## Results

### Terminology

Specialized terminology used throughout the manuscript can be found in Appendix 1.

### Quality assessment

The Supplementary Table 1 shows the results of the STROBE checklist for all included studies. Overall, the quality of the studies was acceptable. The articles had a clear explanation of their scientific background and provided clear explanations of the aims, hypothesis, and experimental design of their study.

### Characteristics of study populations

As shown in Table 1, in total 35 studies were included of which 13 involved people with PD^21–33^, 5 on stroke^34–38^, 9 on TBI^39–47^, 4 on MS^48–51^, and 3 on ALS^52–54^. The overall mean age of the studies was 52.25 (SD:14.65) for all patient groups. All studies report on ERP measures (amplitude and latency), mainly the P3. However, when focusing on the P3, different time-windows were applied ranging from 200 to 700ms after stimulus representation as shown in Table 2.

**Table 1:** Descriptive information of the studies.

Article	Group	Male/Female	Mean age (SD)
Ament, P. A., et al. (1995)	Spinal cord injury	Unknown	19–66 (13.2)
	HC	Unknown	19–66 (13.2)
Bodiswollner, I., et al. (1995)	Parkinson	13F, 17M	61.4 (9.9)
	HC	NA	NA
Cavanagh, J. F., et al. (2018)	Parkinson	9F, 16M	69.68 (8.73)
	HC	9F, 16M	69.32 (9.58)
Ebmeier, K. P., et al. (1992)	Parkinson	7F, 9M	69 (9.2)
	HC	7F, 9M	67 (9)
Georgiev, D., et al. (2015)	Parkinson	6F, 8M	60.39 (12.25)
	HC	6F, 7M	57 (8.58)
Green, J., et al. (1996)	Parkinson	Unknown	54.05 (4.7)
	HC	Unknown	53.9 (3.5)
Iijima, M., et al. (2000)	Parkinson	11F, 9M	63.1 (10.4)
	HC	26F, 29M	60.5 (10.6)
Lagopoulos, J., et al. (1998)	Parkinson	6F, 9M	60.1 (10.2)
	HC	25F, 25M	52.1 (34–60)
Lopes, M. D., et al. (2014)	Parkinson	20F, 24M	64.5 (10.1)
	HC	28F, 5M	65 (54–74)
Rumbach, L., et al. (1993)	Parkinson	14F, 12M	62 (8.1)
	HC	14F, 12M	62 (8.1)
Stanzione, P., et al. (1998)	Parkinson	20F, 24M	60.7 (10.1)
	HC	14F, 17M	55.5 (7.1)
Uslu, A., et al. (2020)	Parkinson	Unknown	41.1 (8.8)
	HC	Unknown	47.5 (8.8)
Vieregge, P., et al. (1994)	Parkinson	3F, 11M	61 (7)
	HC	7F, 9M	61 (8)
Weber, J., et al. (2021)	Parkinson	7F, 6M	71.3 (4)
	HC	8F, 3M	69.4 (6.3)
Ehlers, M. R., et al. (2015)	Stroke	25M, 22F	66.7 (10.4)
	HC	NA	NA
Dejanovic, M., et al. (2015)	Stroke	33F, 27M	57.1 (7.2)
	HC	18F, 12M	56.2 (6.3)
Hirata, K., et al. (1996)	Stroke	Unknown	67.9 (10.6)
	HC	Unknown	66.8 (10.6)
Hsu, L. C., et al. (2018)	Stroke	4F, 10M	55.93 (5)
	HC	10F, 16M	41.83 (2.5)
Yamagata, S., et al. (2004)	Stroke	8F, 21M	71.7 (9.4)
	HC	NA	NA
Doi, R., et al. (2007)	TBI	5F, 14M	33.3 (11.8)
	HC	16F, 16M	33.5 (9.5)
Duncan, C. C., et al. (2003)	TBI	8F, 8M	36.6 (11.8)
	HC	8F, 8M	36.6 (10.4)
Duncan, C. C., et al. (2005)	TBI	5F, 6M	Unknown
	HC	8F, 8M	Unknown
Lew, H. L., et al. (2009)	TBI	2F, 9M	25 (18–49)
	HC	1F, 10M	Unknown
Naito, Y., et al. (2005)	TBI	2F, 38M	48.3 (13.6)
	HC	2F, 38M	48.3 (13.6)
Reinvang, I., et al. (2000)	TBI	28F, 24M	32.8 (10.7)
	HC	28F, 24M	32.8 (10.7)
Reza, M. F., et al. (2007)	TBI	8F, 23M	30.6 (12.9)
	HC	2F, 8M	34.9 (7.1)
Sivak, S., et al. (2008)	TBI	9F, 22M	32 (11.5)
	HC	Unknown	29.7 (11.7)
Unsal, A. and S. J. Segalowitz (1995)	TBI	4F, 16M	31.8 (9.3)
	HC	6F, 16M	32.5 (7.8)
Giesser, B. S., et al. (1992)	MS	9F, 3M	36.5 (9.5)
	HC	4F, 3M	32 (5)
Newton, M. R., et al. (1989)	MS	16F, 7M	37.6 (26–58)
	HC	NA	NA
Triantafyllou, N. I., et al. (1992)	MS	15F, 31M	35.7 (10.2)
	HC	11F, 13M	34.4 (9.4)
Whelan, R., et al. (2010)	MS	16F, 16M	43.82 (8.5)
	HC	15F, 19M	40.11 (9.92)
Ogawa, T., et al. (2009)	ALS	6F, 13M	67.7 (7.4)
	HC	6F, 13M	64.5 (7.4)
Paulus, K. S., et al. (2002)	ALS	8F, 8M	56.1 (11.4)
	HC	NA	NA
Volpato, C., et al. (2010)	ALS	5F, 20M	54.80 (13.42)
	HC	5F, 12M	57.24 (15.58)

*HC* healthy control.

**Table 2:** Descriptive information of auditory oddball paradigms applied in the included studies.

Article	Stimulus length (msec)	Frequency of deviant sounds (Hz)	Frequency non-of deviant sounds (Hz)	Inter-stimulus-interval (msec)	Reporting method	dB of deviant sounds (dB)	Probability of deviant sounds (%)	Location of acquisition
Ament, P. A., et al. (1995)	100	1000	400	Unknown	Silently counting	70	30	Laboratory—Hospital
Bodiswollner, I., et al. (1995)	40	1500	1000	Unknown	Silently counting	75	10	Laboratory
Ehlers, M. R., et al. (2015)	100	2000	1000	900	Silently counting	80	20	NA
Cavanagh, J. F., et al. (2018)	200	660	400	500—1000	Silently counting	80	15	NA
Dejanovic, M., et al. (2015)	NA	2000	1000	100–200	Button press	90	20	Laboratory
Doi, R., et al. (2007)	100	2000	1000	Mean of 1700	Silently counting	70	20	Sound-attenuated, electrically shielded room
Duncan, C. C., et al. (2003)	100	600	1500	1200—1800	Button press	50	10	Laboratory
Duncan, C. C., et al. (2005)	100	1500	600	Unknown	Button press	50	10	Laboratory
Ebmeier, K. P., et al. (1992)	50	1500	1000	1100	Two rounds: 1: Button-press, 2: Silent counting	65	14,30	NA
Georgiev, D., et al. (2015)	200	1000	500	2500	Silently counting	60	15	Laboratory
Giesser, B. S., et al. (1992)	200	1500	500 and 550	2500	Button-press	NA	10	NA
Green, J., et al. (1996)	200	2000	1000	1500	Button-press	72	14	NA
Hirata, K., et al. (1996)	NA	1000	2000	1500	Button press	80	15	NA
Hsu, L. C., et al. (2018)	100	1200	800	Unknown	Silently counting	Unknown	20	Laboratory
Iijima, M., et al. (2000)	50	2000	1000	1700	Silently counting	70	20	NA
Lagopoulos, J., et al. (1998)	50	1500	1000	1300	Silently counting	80	15	Laboratory
Lew, H. L., et al. (2009)	500	500	1000	Mean of 2110	Silently counting	80	20	Laboratory
Lopes, M. D., et al. (2014)	NA	2000	1000	Unknown	Silently counting	80	20	Laboratory
Naito, Y., et al. (2005)	500	2000	1000	1300—1700	Button press	Unknown	20	Laboratory
Newton, M. R., et al. (1989)	50	2000	1000	1200	Button press	Unknown	70	NA
Ogawa, T., et al. (2009)	100	2000	1000	1500	Silently counting	80	20	Sound-attenuated and dimly lit Faraday room
Paulus, K. S., et al. (2002)	150	2000	1000	1300	Silently counting	80	20	Sound attenuating, dimly lit chamber
Reinvang, I., et al. (2000)	50	1200	800	1500	Button-press	80	20	NA
Reza, M. F., et al. (2007)	100	2000	1000	Unknown	Silently counting	60	20	NA
Rumbach, L., et al. (1993)	100	2000	1000	Unknown	Silently counting	60	20	NA
Sivak, S., et al. (2008)	100	2000	1000	1250	Silently counting	70	40	NA
Stanzione, P., et al. (1998)	50	2000	250	1500—2000	Silently counting	70	20	Partially sound-proofed room
Triantafyllou, N. I., et al. (1992)	40	2000	1000	1100	Silently counting	70	20	NA
Unsal, A. and S. J. Segalowitz (1995)	110	1000	1500	1300	Silently counting	60	22,20	NA
Uslu, A., et al. (2020)	50	1500	1000	2000	Button press	70	20	Electrically shielded, sound-attenuated, and dimly lit room
Vieregge, P., et al. (1994)	60	2000	1000	1500	Button press	Unknown	14	NA
Volpato, C., et al. (2010)	400	2000	2000	1000	Silently counting	70	Unknown	Sound attenuated room
Weber, J., et al. (2021)	NA	2000	1000	NA	Button press	Unknown	20	Sound attenuating room
Whelan, R., et al. (2010)	NA	1000	500	2000	Button press	Unknown	20	Soundproofed room
Yamagata, S., et al. (2004)	100	2000	1000	1000—1300	Button press	Unknown	15	Sound-attenuated room

All studies but three, compared the patient group with healthy controls based on neurophysiological measures (ERPs) and neuropsychological measures (cognitive outcome measure). When a healthy control group was included, they were age-matched to the patient group.

### Neuropsychological test results

A variety of neuropsychological tests were used across studies. An overview of all these tests can be found in supplementary Table 2. Not all studies compared neuropsychological test results statistically between healthy controls and the patient groups. When a comparison was made, significant results were found for persons with PD on cognitive screening^23,26,31,33^, verbal fluency^21,25,30,33^, visuospatial skills^21^, visual memory^23^, recognition abilities^23^, intelligence screening^25^, working memory^33^ and sustained attention^30^, indicating better scores for healthy controls. For persons with stroke, significant impairments were found in cognitive screening^36,37^, verbal fluency^38^ and working memory^38^. For persons with ALS, a lower score compared to healthy controls was found for cognitive screening^52–54^, verbal fluency^53,54^, intelligence screening^52^, visual attention^53^ and working memory^54^. For TBI, lower scores were found for intelligence screening^43,44^ and working memory^44^. Last, for MS, only significant results are found for visual memory^48^.

Overall, the results indicate that cognitive screening, verbal fluency and working memory are the cognitive functions that were most impaired within the neurological populations included in this review.

### Experimental paradigm

All experiments applied an auditory oddball paradigm. The mean length of auditory stimuli was 135 ms ranging from 40 ms to 500 ms. The frequency of the deviant sound also varied between studies ranging from 500 Hz to 2000 Hz (Mean: 1604.57 Hz) with 2000 Hz as the frequency used in 41% of the studies. The difference between the deviant and frequent sounds frequency ranged from 500Hz up to 1750 Hz with an inter-stimulus-interval of an average of 1461.48 ms. However, we should note that inter-stimulus-interval was not always reported in all studies. Of all studies included, 21 studies (61.76%) instructed participants to mentally count the number of deviant sounds and report them after each trial. While 14 studies (41.18%) instructed participants to press a button when a deviant sound was presented. The mean (decibels) dB used in all studies was 71.19 dB with SD = 9.84. The probability of the deviant sounds ranged from 10 to 30% with 20% as most used in the included studies. For most studies, participants were instructed to sit silently on a chair and to keep head movements as minimal as possible to control for muscle artifacts. An overview of all descriptive information regarding the paradigm can be found in Table 2.

### Neurophysiological results quantified by the EEG recordings

**Figure 2:**
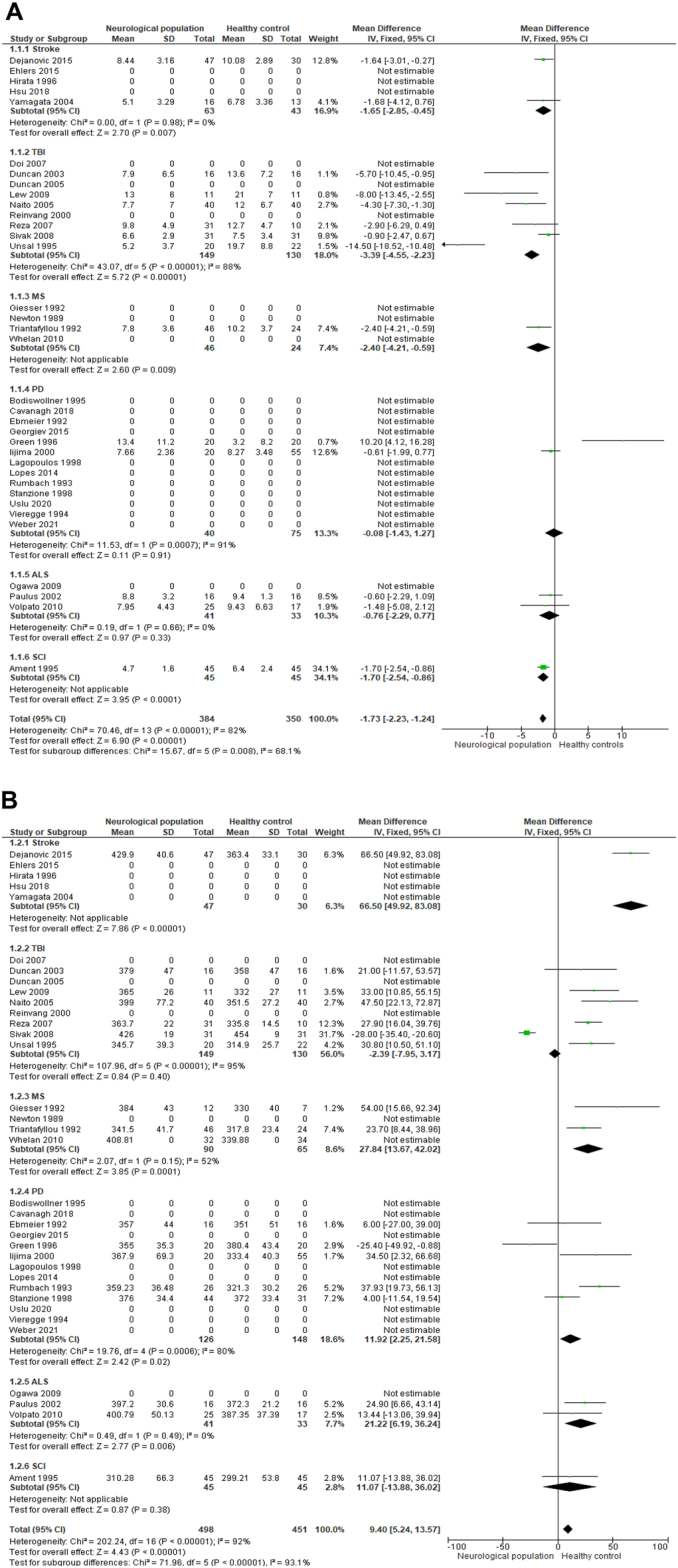
(**A**) Forest plot of random effects for amplitude outcomes. (**B**) Forest plot of random effects for latency outcomes.

Below, we describe P3 amplitude and latency differences between healthy controls and neurological populations presented as a meta-analysis. Forest plots for random-effects meta-analysis stratified by neurological population comparing amplitude and latency outcomes between neurological populations and healthy controls and for all studies combined are presented in Fig. 2. Noteworthy, the meta-analysis of the P3 amplitude contained only one study for MS^50^ and SCI^55^, two studies for stroke^35,38^ and PD^25,26^ and six studies for TBI^40,42,43,45–47^. The meta-analysis of P3 latency included one study for stroke^55^ and SCI^35^, two for ALS^53,54^, three for MS^48,50,51^, five for PD^23,25,26,29,30^ and six for TBI^40,42,43,45–47^.

As seen in Fig. 2A, lower P3 amplitudes were found when comparing all neurological populations to healthy controls (*p* < 0.00001) (mean difference -1.73 with 95 CI -2.23 to -1.24). As seen in Fig. 2B, longer P3 latencies were found when comparing neurological populations to healthy controls (*p* < 0.00001) (mean difference 9.40 with 95% CI 5.24 to 13.57).

Additionally, we compared P3 amplitude and latency of each neurological population separately with healthy controls. The results showed that:

- Lower P3 amplitudes were found for the following neurological populations compared to healthy controls: persons with stroke (*p* = 0.007) (mean difference -1.65 with 95% CI -2.85 to -0.45)), persons with TBI (*p* < 0.00001) (mean difference -3.39 with 95% CI -4.55 to -2.23), persons with MS (*p* = 0.009) (mean difference -2.40 with 95% CI -4.21 to -0.59) and persons with SCI (*p* < 0.0001) (mean difference -1.70 with 95% CI -2.54 to -0.86). However, no significant differences was observed for P3 amplitude in persons with PD as compared to healthy controls (*p* = 0.91) (mean difference -0.08 with 95% CI 1.43 to 1.27).
- Longer P3 latencies were found for the following neurological populations compared to healthy controls: persons with stroke (*p* < 0.00001) (mean difference 66.50 with 95% CI 49.92 to 83.08), MS (*p* < 0.00001) (mean difference 27.84 with 95% CI 13.67 to 42.02) and persons with ALS (*p* = 0.008) (mean difference 21.22 with 95% CI 6.19 to 36.24). However, no significant differences were observed for P3 latency in persons with TBI (*p* = 0.40) (mean difference -2.39 with 95% CI − 7.95 to 3.17), persons with PD (*p* = 0.02) (mean difference 11.92 with 95% CI 2.25 to 21.58) and persons with SCI (*p* = 0.38) (mean difference 11.07 with 95% CI − 13.88 to 36.02).

**Figure 3:**
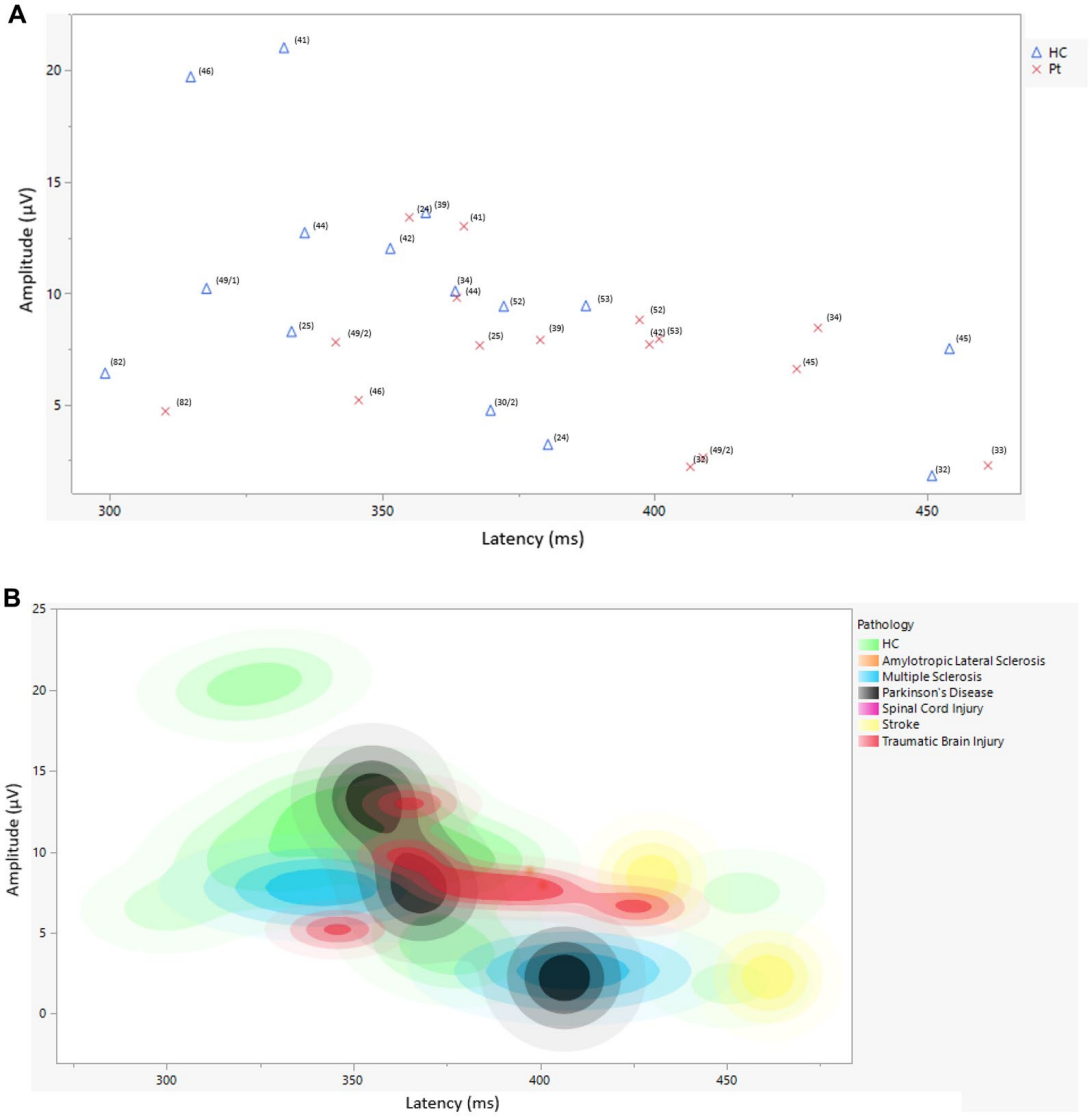
(**A**) Mean amplitude and latency collapsed across studies reporting on these measures, divided by health controls and neurological populations. *All references are indicated at each datapoint using the reference number listen in the reference list. HC = healthy controls. (**B**) Mean amplitude and latency collapsed across studies reporting on these measures, divided by health controls and different neurological populations. *HC healthy controls.

A visual illustration of the mean amplitude and latency collapsed across the different populations can be found in Fig. 3, and a complete overview of P3 amplitude and latency values and the analysis time-windows can be found in Table 3.

**Table 3:** Preprocessing of ERPs and results.

Article	Group	Mean P3 amplitude (SD)	Mean P3 latency (SD)	Amplitude difference	Latency difference	Time-window
Ament, P. A., et al. (1995)	Spinal cord injury	4.70 (1.6)	310.28 (66.3)	HC > PT	HC < PT	250—500
	HC	6.40 (2.4)	299.21 (53.8)			
Bodiswollner, I., et al. (1995)	Parkinson	Unknown	365 (68)	Unknown	HC < PT	200—650
	HC	Unknown	Unknown			
Cavanagh, J. F., et al. (2018)	Parkinson	Unknown	Unknown	HC < PT	Unknown	325 -375
	HC	Unknown	Unknown			
Ebmeier, K. P., et al. (1992)	Parkinson	Unknown	357 (44)	Unknown	NS	280—490
	HC	Unknown	351 (51)			
Georgiev, D., et al. (2015)	Parkinson	Unknown	Unknown	NS	NS	200—700
	HC	Unknown	Unknown			
Green, J., et al. (1996)	Parkinson	13.4 (11.2)	355 (35.3)	HC < PT	NS	250—500
	HC	3.2 (8.2)	380.4 (43.4)			
Iijima, M., et al. (2000)	Parkinson	7.66 (2.36)	367.9 (69.3)	NS	HC < PT	250—500
	HC	8.27 (3.48)	333.4 (40.3)			
Lagopoulos, J., et al. (1998)	Parkinson	Unknown	Unknown	NS	NS	280—550
	HC	Unknown	Unknown			
Lopes, M. D., et al. (2014)	Parkinson	Unknown	344	Unknown	HC < PT	Unknown
	HC	Unknown	Unknown			
Rumbach, L., et al. (1993)	Parkinson	Unknown	359.23 (36.48)	Unknown	HC < PT	250—700
	HC	Unknown	321.3 (30.2)			
Stanzione, P., et al. (1998)	Parkinson	Unknown	376 (34.4)	NS	NS	250—480
	HC	Unknown	372 (33.4)			
Uslu, A., et al. (2020)	Parkinson	Unknown	Unknown	HC > PT	HC < PT	230—420
	HC	Unknown	Unknown			
Vieregge, P., et al. (1994)	Parkinson	Unknown	Unknown	HC > PT	NS	Unknown
	HC	Unknown	Unknown			
Weber, J., et al. (2021)	Parkinson	2.21	406.5	HC < PT	Unknown	300—800
	HC	1.8	450.8			
Ehlers, M. R., et al. (2015)	Stroke	2.27 (1.65)	461.06 (89.22)	HC > PT	Unknown	280—700
	HC	Unknown	Unknown			
Dejanovic, M., et al. (2015)	Stroke	8.44 (3.16)	429.9 (40.6)	HC > PT	HC < PT	250–450
	HC	10.08 (2.89)	363.4 (33.1)			
Hirata, K., et al. (1996)	Stroke	Unknown	Unknown	HC > PT	NS	300—650
	HC	Unknown	Unknown			
Hsu, L. C., et al. (2018)	Stroke	Unknown	Unknown	NS	HC < PT	300—500
	HC	Unknown	Unknown			
Yamagata, S., et al. (2004)	Stroke	5.10 (3.29)	Unknown	Unknown	Unknown	300—600
	HC	6.78 (3.36)	Unknown			
Doi, R., et al. (2007)	TBI	Unknown	Unknown	HC > PT	NS	250—500
	HC	Unknown	Unknown			
Duncan, C. C., et al. (2003)	TBI	7.9 (6.5)	379 (47)	NS	HC < PT	275–575
	HC	13.6 (7.2)	358 (47)			
Duncan, C. C., et al. (2005)	TBI	Unknown	Unknown	NS	HC < PT	Peak at 425
	HC	Unknown	Unknown			
Lew, H. L., et al. (2009)	TBI	13 (6)	365 (26)	HC > PT	HC < PT	270—600
	HC	21 (7)	332 (27)			
Naito, Y., et al. (2005)	TBI	7.7 (7)	399 (77.2)	HC > PT	HC < PT	280—550
	HC	12 (6.7)	351.5 (27.2)			
Reinvang, I., et al. (2000)	TBI	Unknown	Unknown	HC > PT	HC < PT	250—500
	HC	Unknown	Unknown			
Reza, M. F., et al. (2007)	TBI	9.8 (4.9)	363.7 (22)	HC > PT	HC < PT	250—600
	HC	12.7 (4.7)	335.8 (14.5)			
Sivak, S., et al. (2008)	TBI	6.6 (2.9)	426 (19)	NS	NS	Unknown
	HC	7.5 (3.4)	454 (9)			
Unsal, A. and S. J. Segalowitz (1995)	TBI	5.2 (3.7)	345.7 (39.3)	HC > PT	HC < PT	280—500
	HC	19.7 (8.8)	314.9 (25.7)			
Giesser, B. S., et al. (1992)	MS	Unknown	384 (43)	Unknown	HC < PT	250—425
	HC	Unknown	330 (40)			
Newton, M. R., et al. (1989)	MS	Unknown	Unknown	Reduction in amp with age	Unknown	after 270
	HC	5.3	363			
Triantafyllou, N. I., et al. (1992)	MS	7.8 (3.6)	341.5 (41.7)	HC > PT	HC < PT	250—450
	HC	10.2 (3.7)	317.8 (23.4)			
	MS	2.63	408.81	HC > PT	Unknown	Unknown
	HC	4.74	369.88			
Ogawa, T., et al. (2009)	ALS	5.08 (1.8)	370 (29)	Unknown	HC < PT	Unknown
	HC	Unknown	Unknown			
Paulus, K. S., et al. (2002)	ALS	8.8 (3.2)	397.2 (30.6)	NS	HC < PT	after 270
	HC	9.4 (1.3)	372.3 (21.2)			
Volpato, C., et al. (2010)	ALS	7.95 (4.43)	400.79 (50.13)	NS	NS	270—500
	HC	9.43 (6.63)	387.35 (37.39)			

*HC* healthy control.

## Discussion

The aim of this systematic review was to investigate auditory attention differences between neurological populations and healthy controls. Consistent with literature, the studies included in this review applied the auditory-oddball paradigm for these investigations, as the P3 ERP component is frequently used to investigate attentional resources^19^.

Our results show an overall longer P3 latencies and lower amplitudes for neurological populations compared to healthy controls. When comparing each neurological population, we saw that this overall effect in terms of amplitude was present for persons with stroke, TBI, MS and SCI, indicating lower amplitudes for these neurological populations compared to healthy controls. However, this was not the case for PD and ALS. In terms of latency, the overall effect was seen for stroke, MS, PD and ALS indicating longer latencies for the latter neurological populations compared to healthy controls. However, this effect was not seen for TBI and SCI.

The amplitude of the P3 is proportional to the level of attentional resources activated in the processing a stimulus^17^, and in our study, this is specific to the auditory stimulus. The P3 has been reported to be decreased in the presence of attentional deficits^56,57^. The P3 latency reflects the time needed for stimulus evaluation^58^. When latencies are longer, more time is needed to evaluate and process the stimulus^59^. Noteworthy, some factors could influence P3 amplitude and latency such as stimulus significance^60^, global target probability^61–65^, inter-stimulus-interval (ISI)^66,67^, the time-window used^68^ and task-instruction^69^. These are some important aspects to consider when looking at the existing literature. The studies included in this review used a time-window ranging from 200 to 700 ms, with the range usually set between 250 and 600ms^70^.

Our results show inconsistencies in terms of P3 amplitude and latency across neurological populations, mainly in the pathologies of ALS and PD. These results can be explained by either, the limited number of studies that could be included in the meta-analysis, or due to the underlying pathophysiology of the diseases. Below, the latter is elaborated for the different neurological populations.

PD is characterized by lesions within the basal ganglia caused by degeneration of dopaminergic neurons^71^. Within this population, studies have shown that the basal ganglia show preferential activation by perception of rhythms with a steady beat without deviations^72^. In terms of P3 amplitude, a systematic review by De Groote and colleagues (2020)^73^ has shown that auditory perception deficits seen in PD attribute to the impaired central auditory processing; however, sample size and the similarity between deviant sounds and frequent sounds could largely affect results. Additionally, studies show that persons with PD show impaired timing of isochronous intervals^74^ causing the perception of oddballs or changes in rhythms to be impaired.

ALS is an idiopathic progressive neurodegenerative disorder that affects nerve cells in the brain and spinal cord^75^. It primarily targets the motor neurons, which are responsible for controlling voluntary muscle movements^75^. However, no clear studies could be found on the processing of deviances in rhythmic sequences for persons with ALS. This could be explained by the pathophysiology of the disorder as it largely affects motor neurons responsible for muscle control and movement, rather than sensory processing areas of the brain which could explain the lack of differences between persons with ALS and healthy controls.

In persons with TBI, perception of deviances in rhythmic sequences can be impaired as a result of the alteration in brain function due to the trauma caused by an external force^76^. Greater impairment in rhythmic perception is seen for patients with right hemisphere damage compared to the left hemisphere^76^. However, lesion location can highly impact possible processing difficulties of sounds.

In persons with stroke, studies have shown impaired rhythmic perception^77^. This is not always the case and is influenced on the location of the stroke-related lesions. More impairments with rhythmic perception difficulties are reported when damage is found in the basal ganglia and supplementary-motor-area^77^. Evidence suggests a relation between the stroke lesion and acquired amusia, indicating that the ability to perceive rhythms can be impaired within this population^78^.

In persons with MS, an overall consensus could be seen in terms of lower P3 amplitudes and longer P3 latencies compared to healthy controls. Impaired information processing capacities within this population due to impaired connectivity between critical brain regions caused by demyelination is often reported. Studies have shown that up to 50% of persons with MS experience difficulties with information processing^79^. However, evidence shows the capability of persons with MS to synchronize their steps to beats in music and metronomes at different tempi^4^.

The results of this review provide insights that auditory processing is present but impaired in the neurological populations compared to healthy controls: both in terms of magnitude (amplitude) and delay (latency). These insights should be considered when composing the auditory stimuli in strategies using sensorimotor synchronization. For example, considerations of the tempi are required: too fast or too slow tempi would hamper the auditory processing in the presence of the impairment. Another aspect when considering these impairments is the application of adaptive rhythmic systems. Studies have shown that an alignment strategy that continuously adapted the music to the participants' walking pattern showed the best results in terms of synchronization^80^, and these effects have been shown to be favorable in persons with PD as well^81^. Thus, the delay in attentional processes of individual participants need to be considered when developing such alignment strategies. Building on the theme of adaptation, we hereby address the recent development of methodologies designed to capture the dynamic nature of attending^7,82^. In particular, measuring variations in the frequency of oscillatory brain components attuned to the rhythmic stimulus has the potential for future fundamental research on the clinical populations investigated in the present work^83,84^. Among these developments, we point at event-related frequency adjustments (ERFAs) as a viable alternative to traditional ERPs paradigms, to investigate how different pathologies selectively impair oscillatory dynamics underlying auditory attention and sensorimotor synchronization (for details on the experimental paradigm, see^84^).

The impact of designing the stimuli to fit the individual attentional capacities can be seen in anticipating the provision of precision medicine with heightened benefits in terms of longer training durations, or training at higher intensities.

## Limitations

The amount of studies included in this systematic review both reporting on amplitude and latency measures is rather limited, and thus the meta-analysis included a limited number of studies. Within the included studies, no differences were made between P3a and P3b components, making the interpretation of novelty and habituation difficult. Additionally, further sensitivity analysis on the effect of task instruction (i.e. mental counting or button-pressing), on P3 amplitude and latency could not be performed as well. However, studies have shown that motor responses can occlude P3 differences resulting in smaller P3 amplitudes and shorter P3 latencies^69^. Building on the concept of embodied cognition, defined as the body’s interactions with the environment that contribute to cognition^85^, where a motor action—here a button-press—can offload cognitive processing and thus facilitate it. On the other hand, mentally counting the deviant sounds adds a layer of attention and working memory to the task, which might make the task more cognitively difficult compared to a button press, possibly resulting in longer processing times^86^. Additionally, studies did not all report on the cognitive or motor characteristics (or impairments) of the included participants, and thus, these factors could not be assessed within our investigations. Last, the studies included in this review focus on the processing of auditory deviations in rhythmic sequences to better understand how possible processing delays can impact auditory stimulation in rehabilitation settings. However, one could consider that higher order auditory processing is not accounted for (e.g., dichotic listening tasks), where a person is asked to selectively shadow or repeat information presented in one ear while ignoring information presented in the other ear to understand right or left ear advantage^87,88^. To move forward in understanding higher order auditory processing differences between neurological populations and healthy controls, a thorough review of this literature is needed. Further, the current review does not consider the robustness of auditory object formation needed to correctly attend and differentiate between target and non-target auditory stimuli^89^. This could have important implications as the evolution of a sound can impact auditory processing and lead to differences in P3 latency and amplitude, rather than being the result of a neurological condition.

## Conclusion

Overall, neurological populations showed impairments in auditory processing in terms of magnitude (P3 amplitude) and delay (P3 latency) during auditory oddball paradigms compared to healthy controls. Discrepancies in the direction of change of P3 amplitude and latency was found only in persons with PD and ALS for amplitude and in PD and TBI for latency when compared across the neurological pathologies.

Consideration of individual auditory processes and thereafter selecting and/or designing the auditory structure during sensorimotor synchronization paradigms in neurological physical rehabilitation is recommended.

### Supplementary Information

Supplementary Information.

## Data Availability

The datasets analyzed during the current study are available from the corresponding author on reasonable request.
