# Supplementary material for: Auditory attention measured by EEG in neurological populations: systematic review of literature and meta-analysis
Source: Sci Rep. 2023 Nov 29;13:21064. doi: 10.1038/s41598-023-47597-5 (PMC10687139; doi:10.1038/s41598-023-47597-5)
Supplement: Supplementary file 1 — Supplementary Information. [file 41598_2023_47597_MOESM1_ESM.docx]

**Supplemental material**

Table 1. *STROBE checklist.*

| **STROBE statement** | **1** | **2** | **3** | **4** | **5** | **6** | **7** | **8** | **9** | **10** | **11** | **12** | **13** | **14** | **15** | **16** | **17** | **18** | **19** | **20** | **21** | **22** |
| --- | --- | --- | --- | --- | --- | --- | --- | --- | --- | --- | --- | --- | --- | --- | --- | --- | --- | --- | --- | --- | --- | --- |
| Ament, P. A., et al. (1995). | NY | Y | Y | N | Y | YN | Y | Y | N | N | Y | YYNYN | NNN | YN | Y | YYN | Y | Y | N | Y | Y | N |
| Bodiswollner, I., et al. (1995). | NY | Y | Y | Y | N | YN | Y | Y | N | N | N | YYNNN | NNN | YN | Y | YNN | Y | Y | N | Y | Y | Y |
| Ehlers, M. R., et al. (2015). | NY | Y | Y | Y | N | YN | Y | Y | N | N | Y | YYNNN | NNN | YN | Y | YYN | N | Y | Y | Y | Y | N |
| Cavanagh, J. F., et al. (2018). | NY | Y | N | Y | Y | YY | Y | Y | N | N | Y | YYNYN | NNN | YN | Y | YYN | Y | Y | N | Y | Y | Y |
| Dejanovic, M., et al. (2015). | NY | Y | Y | Y | N | YY | Y | Y | N | N | Y | YNNYN | NNY | YN | Y | YYN | N | Y | N | Y | Y | N |
| Doi, R., et al. (2007). | NY | Y | Y | Y | N | YY | Y | Y | N | N | Y | YYNYN | NNN | YN | Y | YYY | Y | Y | N | Y | Y | N |
| Duncan, C. C., et al. (2003). | NY | Y | Y | Y | Y | YY | Y | Y | N | N | Y | YYNYN | YYY | YN | Y | YYY | N | Y | Y | Y | Y | N |
| Duncan, C. C., et al. (2005). | NY | Y | Y | Y | Y | YY | Y | Y | N | Y | Y | YYNYN | NNN | YN | Y | YYY | Y | Y | N | Y | Y | N |
| Ebmeier, K. P., et al. (1992). | NY | Y | Y | Y | Y | YY | Y | Y | N | N | Y | YNNNN | YYN | YN | Y | YNN | N | Y | Y | N | Y | N |
| Georgiev, D., et al. (2015). | NY | Y | Y | Y | Y | YY | Y | Y | N | N | N | YYNYN | YNN | Y | Y | YYY | Y | Y | Y | Y | Y | Y |
| Giesser, B. S., et al. (1992). | NY | Y | Y | Y | Y | YY | N | Y | N | Y | N | YNNNN | NNN | YY | Y | YNN | N | Y | N | Y | Y | Y |
| Green, J., et al. (1996). | NY | Y | Y | Y | N | YY | Y | Y | N | N | N | YYNYN | NNN | YN | Y | YNN | Y | Y | N | Y | Y | Y |
| Hirata, K., et al. (1996). | NY | Y | Y | Y | N | YN | Y | Y | N | N | N | YNNNN | NNN | NN | Y | YNN | N | Y | N | Y | N | N |
| Hsu, L. C., et al. (2018). | NY | Y | Y | Y | N | YY | Y | Y | N | N | Y | YNNNN | NNN | YN | Y | YNY | Y | Y | N | Y | Y | N |
| Iijima, M., et al. (2000). | NY | Y | Y | Y | YY | YY | Y | Y | N | N | N | YYNYN | NNN | YY | Y | YNN | N | Y | N | Y | Y | N |
| Lagopoulos, J., et al. (1998). | NY | Y | Y | Y | Y | YY | Y | Y | N | N | Y | YNNYN | NNN | YN | Y | YYN | Y | Y | N | Y | Y | N |
| Lew, H. L., et al. (2009) | NY | Y | Y | Y | N | YY | Y | Y | N | Y | Y | YNNYN | YNN | YN | Y | YNN | N | Y | N | Y | Y | N |
| Lopes, M. D., et al. (2014). | NY | Y | Y | Y | N | YN | N | Y | N | N | N | YYNYN | NNN | YN | N | YNN | N | Y | N | Y | Y | N |
| Naito, Y., et al. (2005). | NY | Y | Y | Y | N | YY | Y | Y | Y | N | N | YNNYN | NNN | YN | Y | YNN | N | Y | N | Y | Y | N |
| Newton, M. R., et al. (1989). | NY | Y | N | Y | Y | YY | Y | Y | N | N | N | YNNYN | NNN | YN | Y | YNN | N | Y | N | Y | Y | Y |
| Ogawa, T., et al. (2009). | NY | Y | Y | Y | N | YY | Y | Y | N | N | Y | YNNYN | YNN | YN | Y | YNY | N | Y | N | Y | Y | N |
| Paulus, K. S., et al. (2002). | NY | Y | Y | Y | N | YY | Y | Y | N | N | N | YNNYN | YNN | YN | Y | YYN | N | Y | N | Y | Y | N |
| Reinvang, I., et al. (2000). | NY | Y | Y | Y | Y | YY | Y | Y | N | N | Y | YYNYN | YNN | YN | Y | YNN | Y | Y | N | Y | Y | N |
| Reza, M. F., et al. (2007). | NY | Y | Y | Y | Y | YY | Y | Y | N | Y | N | YNNYN | YNN | YN | Y | YYN | N | Y | Y | Y | Y | N |
| Rumbach, L., et al. (1993). | NY | Y | Y | Y | Y | YY | Y | Y | N | N | N | YNNYN | YNN | YY | Y | YNN | N | Y | N | Y | Y | N |
| Sivak, S., et al. (2008). | YY | Y | Y | Y | Y | YY | Y | Y | N | Y | Y | YNNYN | YNN | YN | Y | YYN | N | Y | N | Y | Y | N |
| Stanzione, P., et al. (1998). | NY | Y | Y | Y | Y | YY | Y | Y | N | Y | Y | YYNYN | YNN | YN | Y | YYN | N | Y | N | Y | Y | Y |
| Triantafyllou, N. I., et al. (1992). | NY | Y | N | N | Y | YY | Y | Y | N | Y | Y | YNNYN | YNN | YN | Y | YYN | N | Y | N | Y | Y | N |
| Unsal, A. and S. J. Segalowitz (1995). | NY | Y | Y | Y | Y | YY | Y | Y | N | Y | N | YYNYN | YNN | YN | Y | YNN | N | Y | N | Y | Y | Y |
| Vieregge, P., et al. (1994). | NY | Y | Y | Y | Y | YY | Y | Y | N | Y | N | YYNYN | YNN | YN | Y | YNN | N | Y | N | Y | Y | N |
| Volpato, C., et al. (2010). | NY | Y | Y | Y | Y | Y | YY | Y | Y | N | Y | YYNYN | YNN | YN | Y | YYN | N | Y | N | Y | Y | N |
| Weber, J., et al. (2021). | NY | Y | Y | Y | Y | YY | Y | Y | N | Y | Y | YYNYN | YNN | YN | Y | YYY | N | Y | Y | Y | Y | N |
| Whelan, R., et al. (2010). | NY | Y | Y | Y | Y | YY | Y | Y | N | N | Y | YNNYN | YNN | YN | Y | YYN | Y | Y | N | Y | Y | N |
| Yamagata, S., et al. (2004). | NY | Y | Y | Y | Y | YY | Y | Y | N | Y | Y | YYNYN | YNN | YN | Y | YYN | N | Y | N | Y | Y | Y |

Table 2. *Overview of neuropsychological assessments.*

| Article | Neuropsychological test | Cognitive domain | | Mean of HC (SD) | Mean PT (SD) | |  |
| --- | --- | --- | --- | --- | --- | --- | --- |
| Ebmeier, K. P., et al. (1992)  Hirata, K., et al. (1996)  Hsu, L. C., et al. (2018)  Iijima, M., et al. (2000)  Ogawa, T., et al. (2009)  Paulus, K. S., et al. (2002)  Uslu, A., et al. (2020)  Volpato, C., et al. (2010) | Mini-Mental State Examination (/30) | | Memory screening | 28.6 (1.4)  26.38 (3.89)  *Unknown*  *Unknown*  *Unknown*  26.4 (2.3)  29.8 (0.4)  28.88 (1.37) | 25.7 (3.6)  20.6 (2.66)  *Unknown*  *Unknown*  27.5 (1.6)  24.1 (4.7)  29.6 (0.7)  28.45 (1.68) | |  |
| Bodiswollner, I., et al. (1995)  Green, J., et al. (1996)  Paulus, K. S., et al. (2002)  Weber, J., et al. (2021)  Yamagata, S., et al. (2004)  Stanzione, P., et al. (1998)  Volpato, C., et al. (2010) | COWAT | | Verbal fluency | *Unknown*  *Unknown*  13.2 (4.9)  14.4 (5.1)  11.2 (3.2)  *Unknown*  37.71 (14.92) | 44.4 (12.9)  *Unknown*  7.4 (4.2)  12.2 (3.6)  8.7 (3.0)  *Unknown*  28.91 (8.34) | |  |
| Bodiswollner, I., et al. (1995) | Line Orientation Test | | Visuospatial skills | *Unknown* | 21.6 (6.7) | |  |
| Ebmeier, K. P., et al. (1992)  Giesser, B. S., et al. (1992) | Benton Visual retention Test | | Visual memory | 12.3 (1.8)  2.0 | 10.2 (2.7)  7.0 | |  |
| Ebmeier, K. P., et al. (1992) | Face recognition Test | | Recognition abilities | 48.4 (4.2) | 42.3 (7.6) | |  |
| Green, J., et al. (1996)  Naito, Y., et al. (2005)  Ogawa, T., et al. (2009)  Reinvang, I., et al. (2000) | Wechsler adult Intelligence scale – R: verbal subtests and block design | | Non-verbal reasoning and understanding of abstract visual information – common sense social knowledge – numerical reasoning and mental arithmetic abilities – working memory – verbal fluency – logical thinking – ability to analyze and synthesize abstract designs | 117.4 (8.4)  101.4 (15)  *Unknown*  *Unknown* | 119.2 (11.8)  87.3 (17.7)  98.2 (11)  98 (15) | |  |
| Paulus, K. S., et al. (2002) | Visual Search Test | | Visual attention | 51.2 (8.8) | 42.9 (10) | |  |
| Reinvang, I., et al. (2000)  Volpato, C., et al. (2010)  Weber, J., et al. (2021)  Yamagata, S., et al. (2004) | Digit Span | | Working memory | *Unknown*  5.29 (0.84)  6.5 (1.4)  *Unknown* | 47 (10.7)  5.25 (1.15)  5.2 (1.5)  *Unknown* | |  |
|  |  | |  |  |  | |  |
| Stanzione, P., et al. (1998) | Copying and designs test | | Sustained attention | *Unknown* | *Unknown* | |  |
|  |  | |  |  |  | |  |
| Weber, J., et al. (2021) | MoCA | | Cognitive screening | 26.6 (1.9) | 24.1 (2) | |  |
| *HC = healthy controls |  | |  |  | |  | |

Appendix 1. *Definitions of specialized terminology*

| Sensorimotor synchronization | The alignment of a sensory and a motor systems, for example alignment of footsteps during walking with beats in a song. |
| --- | --- |
| Electroencephalography (EEG) | A method for recording electrical brain activity using surface electrodes. |
| Event-related-potentials (ERPs) | Changes in the ongoing EEG activity in response to a specific event that are time-locked to sensory, motor, and cognitive events. |
| P300 (P3) | Event-related-potential that occurs around 300ms after stimulus representation seen as a proxy to for attentional resources, as it reflects one’s discrimination abilities between the deviant and standard events in the auditory stimuli (Polich., 1987). |
